# Interaction Interface of Aβ_42_ with Human Na,K-ATPase Studied by MD and ITC and Inhibitor Screening by MD

**DOI:** 10.3390/biomedicines10071663

**Published:** 2022-07-11

**Authors:** Alexei A. Adzhubei, Anna P. Tolstova, Maria A. Strelkova, Vladimir A. Mitkevich, Irina Yu. Petrushanko, Alexander A. Makarov

**Affiliations:** Engelhardt Institute of Molecular Biology, Russian Academy of Sciences, 119991 Moscow, Russia; alexei.adzhubei@eimb.ru (A.A.A.); strelkova.ma@phystech.edu (M.A.S.); mitkevich@eimb.ru (V.A.M.); ipetrushanko@eimb.ru (I.Y.P.)

**Keywords:** Alzheimer’s disease, Na,K-ATPase, beta amyloid, interaction interface, interaction inhibitors screening, conformations of Na,K-ATPase, ouabain, isothermal titration calorimetry, binding constants, molecular dynamics

## Abstract

Alzheimer’s disease (AD) is a neurodegenerative disease accompanied by progressive cognitive and memory dysfunction due to disruption of normal electrotonic properties of neurons and neuronal loss. The Na,K-ATPase interaction with beta amyloid (Aβ) plays an important role in AD pathogenesis. It has been shown that Na,K-ATPase activity in the AD brain was significantly lower than those in age-matched control brain. The interaction of Aβ_42_ with Na,K-ATPase and subsequent oligomerization leads to inhibition of the enzyme activity. In this study interaction interfaces between three common Aβ_42_ isoforms, and different conformations of human Na,K-ATPase (α1β1) have been obtained using molecular modeling, including docking and molecular dynamics (MD). Interaction sites of Na,K-ATPase with Aβ_42_ are localized between extracellular parts of α- and β- subunits and are practically identical for Na,K-ATPase at different conformations. Thermodynamic parameters for the formation of Na,K-ATPase:Aβ_42_ complex at different conformations acquired by isothermal titration calorimetry (ITC) are similar, which is in line with the data of molecular modeling. Similarity of Na,K-ATPase interaction interfaces with Aβ in all conformations allowed us to cross-screen potential inhibitors for this interaction and find pharmaceutical compounds that could block it.

## 1. Introduction

Alzheimer’s disease (AD) is a neurodegenerative disease accompanied by progressive cognitive and memory dysfunction due to neuronal loss. At the moment, there is no effective therapy for AD. Despite the fact that AD has been studied for decades, its causes and molecular mechanism of the disease progression are not well understood. A marker for AD is represented by a deposition of Aβ peptide in the brain tissue in the form of insoluble senile plaques [1]. Soluble nonfibrillar aggregates of Aβ peptides have strong neurotoxic effects [2,3,4,5]. Posttranslational modifications of the Aβ_42_ peptide, especially Asp7 isomerization and Ser8 phosphorylation, affect its oligomerization and neurotoxic effect [6,7,8,9,10]. At the same time, Aβ is a regulatory peptide that interacts with different target proteins in a healthy organism, and during AD is involved in pathogenic reactions that result in neurotoxic effects and neurodegeneration in brains of AD patients [11,12,13]. Some researchers insist on the primary role of pathogenic cascades induced by Aβ interaction with its targets (receptors) in AD severity [11,13]. There is evidence that Aβ binds to postsynaptic receptors, such as α7-nicotinic acetylcholine (α7nAChR) [14] and α4β2-nicotinic acetylcholine (α4β2 nAChR) [15], receptors for advanced glycation end products (RAGE) [16], NLRP3 inflammasome [17], Na,K-ATPase [18] and others.

Here we study the binding interface of Aβ with Na,K-ATPase. Na,K-ATPase creates a sodium/potassium gradient, and inhibition of pump activity by Aβ leads to impairment of electrotonic properties of neurons [19]. It was shown that interaction of Aβ with Na,K-ATPase contributes significantly to the development of Alzheimer’s disease [20]. It was also demonstrated that Na,K-ATPase activity in the AD brain was significantly lower than those in age-matched control brains. Na,K-ATPase were also significantly reduced in the AD brain [21]. The data suggest that the reduction in Na,K-ATPase activity in tissue affected by AD may not be purely secondary to neurodegeneration, but may result from direct effects of amyloid on this protein [20]. This hypothesis is confirmed by research on direct Na,K-ATPase interaction with Aβ_42_ [18]. It was shown that the complex formation results in dose-dependent inhibition of enzyme hydrolytic activity. Interaction of Na,K-ATPase with exogenous Aβ_42_ leads to a pronounced decrease in enzyme transport and hydrolytic activity in neuroblastoma cells SH-SY5Y [18]. Enzyme inhibition can occur due to Aβ_42_ oligomerization, when the amyloid molecule is primarily bound to Na,K-ATPase [10]. This is confirmed by data showing that the phosphorylated peptide pS8-Aβ_42_, which has a lower tendency to oligomerization, does not cause inhibition, despite binding to the enzyme [10]. Thus it can be conjectured that blocking Aβ_42_ binding to Na,K-ATPase would prevent its inhibition and reduce the disruption of the electrogenic properties of neurons in AD.

However, the interaction interface between Na,K-ATPase and Aβ_42_ remains unstudied. Na,K-ATPase during the catalytic cycle passes through a series of conformational transitions from E1 to E2 states through the phosphorylated forms E1P and E2P [22]. To establish the interaction interface and search for inhibitors, it was necessary to discover which conformation of Na,K-ATPase amyloid binds to and how the changes in the conformation of the enzyme affect this interaction.

In this study, interfaces for the interaction of Aβ_42_ with Na,K-ATPase in different conformations have been obtained using molecular modeling approaches. We found that the interaction interfaces of Aβ_42_ with different conformations of Na,K-ATPase are practically identical, which was confirmed by close values of thermodynamic parameters of Aβ_42_ binding to different states of Na,K-ATPase, obtained by isothermal titration calorimetry (ITC). The interaction interface did not change significantly for isomerized and phosphorylated isoforms of Aβ_42_ either_._ Then we constructed and applied a novel method for the in silico inhibitor search based on a combination of docking procedures utilizing multiple docking servers, with subsequent docking data refinement by molecular dynamic simulations (MD). According to our results, the prospective inhibitors will be effective for all conformations of Na,K-ATPase.

## 2. Materials and Methods

### 2.1. Structure Preparation for Molecular Modeling

The human α1 Na,K-ATPase structure in the E1P and E2P states and in complex with specific ligands of the Na,K-ATPase cardiotonic steroid ouabain (OBN Na,K-ATPase conformation) was constructed by point mutations of the *sus scrofa* and *squalus acanthias* Na,K-ATPase structures (PDB:3WGU [23], PDB:2ZXE [24] and PDB:4HYT [25], respectively) using UNIPROT sequence P05023 AT1A1_HUMAN for the final structure. These structures were incorporated in the DDPC membrane and submitted for subsequent simulation for 50 ns of molecular dynamics using GROMACS software [26]. The CHARMM-GUI [27] membrane builder was used to construct the system of Na,K-ATPase at different states embedded in the membrane. The position of the membrane was checked according to the UNIPROT data for P05023 (AT1A1_HUMAN).

Structures used as templates for the initial expert modeling of Aβ_42_ were selected from the data of our analysis of Aβ structures in the PDB [28], together with the previously created model of Aβ_42_ [29]. Aβ_42_ modifications with the phosphorylated Ser8 (pS8-Aβ) and isomerized Asp7 (isoD7-Aβ) were constructed by expert modeling. All the structures were equilibrated and relaxed during MD production run in water with the ion concentration of 115 mM.

### 2.2. Protein–Protein Docking and Molecular Dynamic Parameters

Equilibrium structures were used in global full blind and targeted docking using the servers SwarmDock [30], PatchDock [31], and Haddock [32] and in-house software for flexible docking. Isomerized residues are not supported in the above docking software, so we used the equilibrium structure of isoD7-Aβ with standard asparagine and then changed it into iso-Asp7 after docking. Phosphorylated residues are not supported in some docking software either; therefore, for the docking of pS8-Aβ, we used a limited set of servers. All the docking results were analyzed with in-house software QASDOM [33]. and for the docking of pS8-Aβ we used a limited set of servers.

Molecular dynamics of the resulting docked complexes Aβ_42_:Na,K-ATPase was performed using Gromacs software package [26]. The initial structures were subjected to energy minimization with the following consecutively applied algorithms, steepest descent and conjugated gradients. Then they were equilibrated in water with the NaCl concentration of 115 mM under position restraints for 1 ns in the NVT and NPT ensembles, respectively. The CHARMM36 force field was used [34]. Topology for the isomerized asparagine was constructed by modifying the CHARMM36 force field for standard asparagine residue, while the bonded and nonbonded parameters were retained. MD calculations were performed with the particle-mesh Ewald technique with repeating boundary conditions and 1 nm cutoffs. A LINCS constraint algorithm with a 2 fs time step was applied. A constant temperature of 300 K was maintained throughout computations with two coupling and energy groups.

### 2.3. The Molecular Modeling Algorithm

We present a novel algorithm to identify potential inhibitors of protein–protein interaction by computer modeling. In this study, this algorithm was applied to the Aβ_42_–Na,K-ATPase interaction.

**Figure 1:**
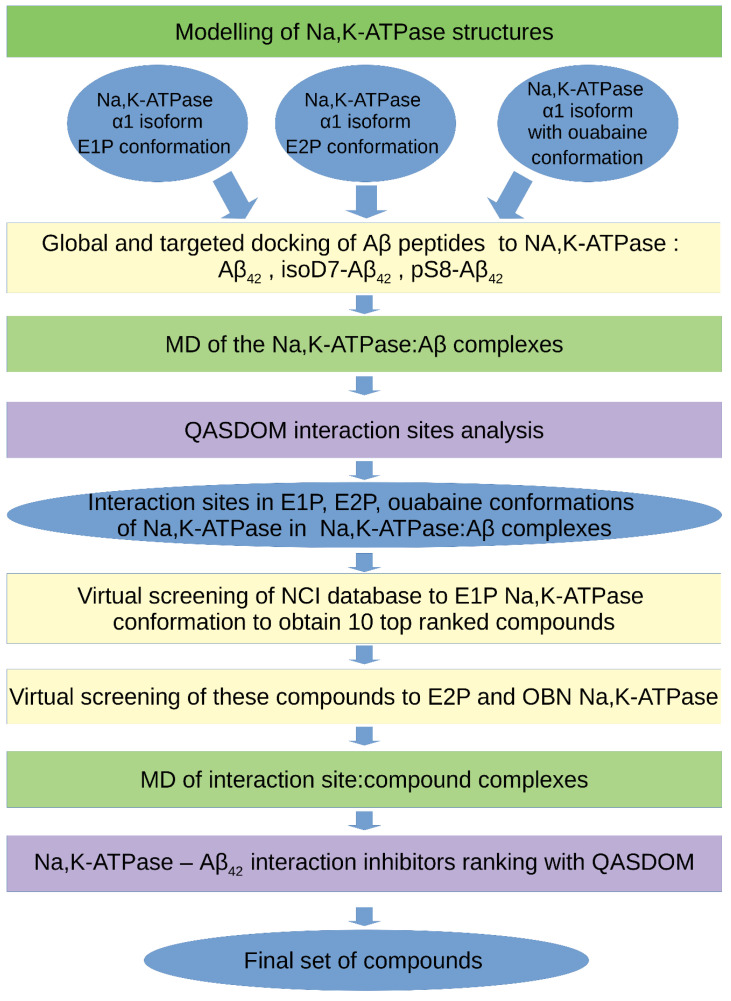
The workflow scheme for the inhibitor search for the Aβ_42_–Na,K-ATPase interaction by computer modeling.

The main steps of the algorithm are shown in Figure 1 and can be described as follows.

1. Obtaining an equilibrium structure of the target protein (in our case, of the three Na,K-ATPase conformations) in complex with a membrane (if necessary) by application of MD simulation.

2. Global targeted docking of the ligands (in our case, three Aβ_42_ isoforms described above) to the target protein (in our case, three Na,K-ATPase conformations described above) using docking servers, i.e., Haddock, SwarmDock and PatchDock.

Although these servers provide, according to our data, the most realistic models of Aβ_42_ complexes with the target proteins in comparison with other similar servers, they do not support the option of a membrane in docking procedures for membrane proteins. Only those complexes that did not overlap the transmembrane region of the target protein were used.

3. Analysis of the intermolecular contacts in the obtained complexes with the QASDOM server. Creating a rating of the complexes by scoring function. Selection of 5–10 complexes with the best affinity according to the QASDOM data for each protein:ligand complex (in our case, five models for each Aβ_42_ isoform, a total of 15 models for each Na,K-ATPase conformation).

4. Molecular dynamics to obtain an equilibrium structure of the selected complexes.

5. Analysis of the intermolecular contacts in the final complexes after MD using the QASDOM server to determine the interaction sites and a specific interaction interface for the target protein.

6. Structure-based virtual screening of drug compounds from the NCI Open Database of chemical compounds (~260,000 compounds) to the identified target protein interface.

7. Molecular dynamics of the top-rated compounds in complexes with a target protein.

8. Analysis of the intermolecular contacts in the final complexes after MD with the QASDOM server. This step is necessary, because during MD, the compound can change its position in space and leave the intended interaction site. Therefore, after MD, the final effect of inhibition can be either higher or lower in comparison with the initial position of the compound after virtual screening.

9. Obtaining a final list of drug compounds that represent potential inhibitors of the target protein–protein interaction.

### 2.4. Na,K-ATPase and Aβ_42_ Preparations for ITC

Aβ_42_ peptide was synthesized by Biopeptide. The preparation of the Aβ_42_ was carried out according to the protocol [35] and as described by us in detail earlier [18]. Briefly, the peptide was dissolved in hexafluoroisopropanol (Fluka), dried, and stored at −80 °C. Before the experiment, the peptide was dissolved in DMSO for 1 h and then diluted in the buffer solution. As we showed earlier, after such preparation, the Aβ_42_ was present in the solution, mainly in the monomeric form [18].

Purified preparation of Na,K-ATPase [36] was obtained from duck salt glands as described elsewhere [37,38]. The purity grade of Na,K-ATPase was 99% and specific activity of the enzyme reached ~2400 μmol of Pi (mg of protein × h)^−1^ at 37 °C.

### 2.5. Isothermal Titration Calorimetry

The thermodynamic parameters of Aβ_42_ and ouabain binding to duck Na,K-ATPase were measured using a MicroCal iTC200 instrument (MicroCal, Northampton, MA), as described elsewhere [18]. Experiments were carried out at 25 °C in four buffers, as described elsewhere [18,39]: (E2P buffer) 10 mM imidazole, 3 mMTris/Pi, 1 mM EDTA, 3 mM MgCl_2_, 0.1 mM DTT, pH 7.5; (E1/E2 buffer) 10 mM imidazole, 130 mM NaCl, 20 mM KCl, 3 mM MgCl_2_, 0.1 mM DTT, pH 7.5; (E1 buffer) 10 mM imidazole, 1 mM EDTA, 3 mM NaCl, 0.1 mM DTT, pH 7.5; (E2 buffer) 10 mM imidazole, 1 mM EDTA, 3 mM KCl, and 0.1 mM DTT, pH 7,5. To determine the effect of ouabain on Aβ_42_ binding, sequential titration of Na,K-ATPase with ouabain was done, followed by titration with Aβ_42_ in E2P buffer (OBN state, Table 2). Similarly, an experiment was carried out to determine the effect of Aβ_42_ on the binding of ouabain. Aliquots (2.6 μL) of ligands were injected into a 0.24 mL cell containing the protein solution to achieve a complete binding isotherm. Protein concentration in the cell ranged from 15 to 20 μM, ouabain concentration in the syringe was equal to 180 μM, and Aβ_42_ concentration in the syringe was equal to 200 μM. Equivalent amounts of DMSO was added to protein solutions to avoid thermal effects of dissolution.

The effective heat of binding was obtained by subtracting the heat of dilution from the heat of reaction. MicroCal Origin software was used to fit isothermal titration curves. As a result, the following thermodynamic binding parameters were obtained: binding constants (K_a_) and enthalpy changes (ΔH). The Gibbs energy and enthalpy changes (ΔS) were estimated according to the equations: ∆G = −RT lnKa and ∆G = ∆H − T∆S.

## 3. Results

### 3.1. Interaction Interface of Na,K-ATPase in Various Conformations with Aβ_42_ and Its Isoforms

Previously, we showed that Aβ_42_ monomers interact with the extracellular part of Na,K-ATPase, and according to the data of Aβ_42_ docking to the extracellular shark Na,K-ATPase crystal structure (PDB: 2ZXE [24]), the binding site of Aβ_42_ was localized in the “gap” between the alpha- and beta-subunits of Na,K-ATPase [18]. The known data on the Na,K-ATPase interaction with Aβ_42_ cannot show the exact interaction area or its interface. Moreover, during the catalytic cycle, Na,K-ATPase passes through two main conformations (E1 and E2), but the pattern of changes in the interaction between Aβ_42_ and Na,K-ATPase it not known yet. To define the interaction interface between Aβ_42_ and Na,K-ATPase, as a first step we have utilized unconstrained full-blind docking of the Aβ_42_ peptide to the human α1 Na,K-ATPase structure at the E1P and E2P conformations, as described in Methods. This was performed to roughly approximate the interaction sites with Aβ_42_ in Na,K-ATPase. From the full set of the resulting complexes, we selected structures that met the following criteria: (1) Aβ_42_ is mainly positioned in the Na,K-ATPase cavity (located between the α- and β-subunits of Na,K-ATPase) or near it, (2) there are no interactions with the membrane part of the protein, (3) and Aβ_42_ interacts with Na,K-ATPase in the multiple points of its structure or with comparatively large area in the protein. The full-blind unconstrained docking provided only five complexes for the E1P conformation of Na,K-ATPase (showed in Figure S1) and two complexes for E2P conformation. All structures of Aβ peptides from these complexes had an elongated shape and were wrapped around the Na,K-ATPase cavity near the membrane surface. According to these data, the Na,K-ATPase surface delimited by the residues 119-AATEEE-124, 311-LILEY-315, and 887-RVDWDDRWIND-897 in the α-subunit and 84-QKTEI-88 in the β-subunit were identified as an interaction interface and a target site for the targeted global docking of Aβ_42_ isoforms to Na,K-ATPase at different conformations.

**Figure 2:**
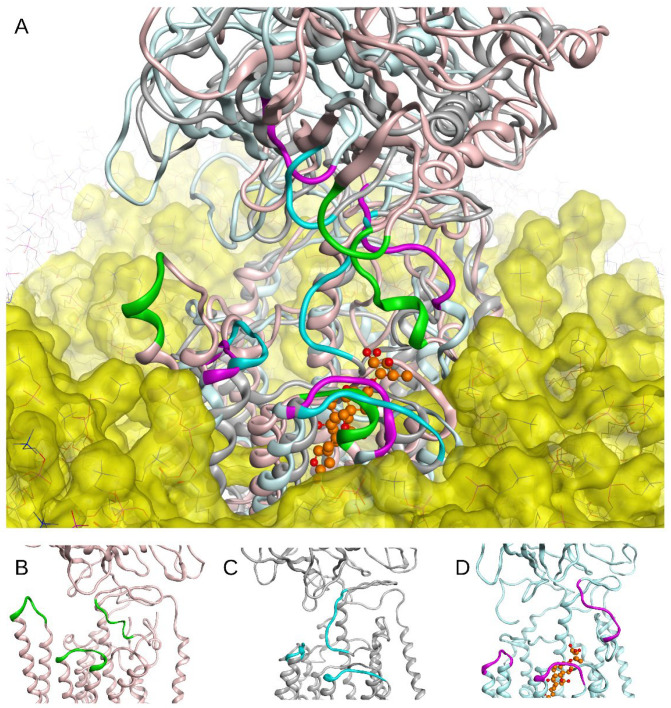
Superposition of Na,K-ATPase E1P, E2P states and Na,K-ATPase in complex with ouabain (OBN) embedded in DDPC membrane after 50 ns of MD (**A**). The Na,K-ATPase structure is shown in (**B**–**D**) for E1P (**B**), E2P (**C**), and OBN (**D**) states. The residues whose positions differed between conformations are shown with green (E1P), cyan (E2P), and magenta (OBN). Ouabain atoms are shown in orange.

**Figure 3:**
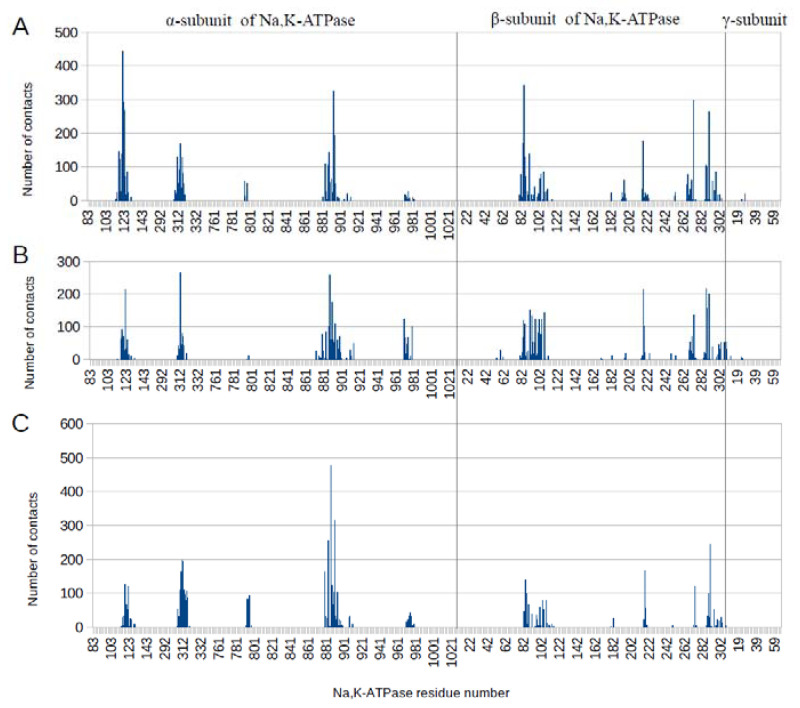
The number of intermolecular contacts with three Aβ isoforms after 30–50 ns of MD in E1P (**A**), E2P (**B**), and OBN (**C**) conformations of α1 isoform of human Na, K-ATPase. The data for each conformation are summarized for 15 complexes that participated in the MD.

Structural alignment of the three different states (E1P, E2P, and OBN) of α1 Na,K-ATPase relaxed by MD simulation showed significant structural differences in the probable interaction area with Aβ_42_ (Figure 2). As a result of these structural differences, the global docking of the three Aβ_42_ isoforms (Aβ_42_, isoD7-Aβ, pS8-Aβ) to the probable interaction sites in Na,K-ATPase showed slightly different contacting residues for different conformations of Na,K-ATPase (Figure S2). To clarify the docking data, the obtained complexes of Na,K-ATPase: Aβ_42_ (five best complexes for each of the three Aβ_42_ variants with each conformation of Na,K-ATPase) were subjected to MD modeling for 30–50 ns. Subsequently, we reanalyzed the contacts in these complexes with QASDOM (Figure 3 and Figure S2). There was no difference in the interaction sites in any of the Na,K-ATPase conformations in complex with different Aβ_42_ isoforms, although the relevant contact numbers differed. We attribute this difference to the small number of models, and therefore it was deemed not significant. The number of contacts was summarized over all three Aβ_42_ isoforms, i.e., 15 models for each Na,K-ATPase conformation were included in the analysis of the interaction with Aβ_42_. The MD results showed little divergence in the interaction regions between different conformations of Na,K-ATPase, less than after docking (Figure 3 and Figure S2). These data are shown in Table 1 and Figure 3. We conclude that the MD data represent refined docking data, where the peaks became narrower. Some areas of interaction were removed from the final model after MD.

According to the 3D arrangement, frequency, and density of the contacts with Aβ_42_ among all conformations of the α1 isoform of Na,K-ATPase, we consider the most promising for inhibition of the interaction of Na,K-ATPase: Aβ_42_ in the following four regions. They are delineated in the α-subunit by residues 119-AATEE-123, 310-SLILEY-315, 887-RVDWDDR-893, and include one region in the β-subunit 84-QKTEI-88 (Figure S3). These areas are located close to each other structurally, and it should be possible to select a universal inhibitor that will block all these sites concurrently. The obtained results on interaction sites of Na,K-ATPase with Aβ_42_ were in agreement with our previous data of shark Na,K-ATPase: Aβ_42_ complexes, in which structurally similar sites Glu88, Ser101, Glu273, and Arg292 were participating in the interaction with Aβ_42_ [18]. Here, we omitted from our analysis a large interaction area located in the β-subunit, including the residues 216-KRDEDKDKVG-226, 267-TMDTEIR-273, and 285-YSEKDR-290 (Figure S3). These residues are located far away from the Na,K-ATPase cavity between α- and β-subunits, and we hypothesize that they do not contribute to the reduction in Na,K-ATPase activity by Aβ_42_ [18].

For Aβ_42_, there was no clear advantage of any single peptide interaction site. Even the orientation of Aβ_42_ was similar (for E1P conformation of Na,K-ATPase in 17 cases, the C-terminus of the peptide was inside the cavity at the entrance to the Na,K-ATPase channel, and in 13 cases the N-terminus of the peptide was inside the cavity). In the majority of docking models, the Aβ_42_ peptide was positioned in parallel with the modeled membrane surface. Directions of the structure packing of the peptides from N- to C-termini and vice versa wrapped around the cavity region of Na,K-ATPase occurred roughly equally.

### 3.2. Binding of Na,K-ATPase at Different Conformations with Aβ_42_

To study the binding of Aβ_42_ to Na,K-ATPase in various conformations, we used ITC. The results included estimated association (Ka) and dissociation (Kd) equilibrium constants, and changes in enthalpy (∆H) and entropy (∆S) for the binding of Aβ_42_ to Na,K-ATPase in five conformations: E1/E2, E1, E2, E2P, and OBN (Table 2).

For Na,K-ATPase in all conformations, the binding stoichiometry of Aβ_42_ to the enzyme was 1:1 and the interaction enthalpy-driven (Table 2). Thermodynamic parameters for complex formation of Na,K-ATPase:Aβ_42_ at different conformations were similar, in line with the data of molecular modeling, which also showed similarity of the molecular interface of Aβ_42_ interaction with the enzyme at different conformations. In complex with ouabain (OBN conformation), Na,K-ATPase interacted with Aβ_42_ in the same way as the free enzyme (Table 2). In complex with Aβ_42_ the binding constant of Na,K-ATPase with ouabain (K_d_ = 0.12 ± 0.03 µM, ∆H = −20.2 ± 0.7, T∆S= −10.8) did not change from the enzyme in E2P conformation (K_d_ = 0.10 ± 0.01 µM, ∆H = −13.7 ± 0.2, T∆S = −4.2).

### 3.3. Virtual Screening of Inhibitors for Na,K-ATPase: Aβ_42_ Interaction

The structure-based virtual screening was performed using the NCI Open Database Compounds (266,151 compounds) to the interaction interface area of E1P Na,K-ATPase:Aβ_42_, specifically to all interaction sites with Aβ_42_ listed above. Virtual screening of such a large database required substantial time and computer resources. To reduce computational costs, it was decided not to screen each conformation of Na,K-ATPase independently, since we were interested in finding inhibitors for the overall Na,K-ATPase:Aβ_42_ interaction interface regardless of Na,K-ATPase conformation, but to dock the ten top-rated compounds from the virtual screening results of E1P to E2P and OBN Na,K-ATPase. These compounds were docked to the same combined interaction interface at E2P Na,K-ATPase, and OBN Na,K-ATPase (Table 3). The resulting ten best complexes with each Na,K-ATPase conformation were also subjected to 50 ns MD simulation to refine docking results.

The MD results of Na,K-ATPase:compound complexes are shown in Table 2. The majority of the ligands shifted from the interaction site. The ligands that continued to efficiently overlap the Aβ_42_ interaction site in Na,K-ATPase after the MD run are highlighted in green in Table 3. In the six screening complexes out of 30 (ten complexes for each Na,K-ATPase conformation), the ligands moved to the membrane as a result of MD. They are highlighted in red in Table 3. Immersion of the ligands in the membrane occurred due to the chemical properties of the ligands and did not depend on the Na,K-ATPase conformation. This was observed for all three Na,K-ATPase conformations for the compound NCI686480 and for two conformations for the compound NCI688806. The compound NCI610512 moved to the membrane in one conformation of Na,K-ATPase, and in the OBN Na,K-ATPase conformation it entered the Na,K-ATPase cavity and therefore has did not block the interaction site. We assume that these ligands had low probability of blocking the Aβ_42_ interaction site in Na,K-ATPase in vitro, and therefore we eliminated NCI688806, NCI610512, and NCI686480 from the final ranking.

In the case of OBN Na,K-ATPase conformation, more ligands remained bound to the interaction interface than the other conformations. However, we observed several occurrences of ligands entering the Na, K-ATPase channel. These cases are highlighted in yellow in Table 3. We assumed that these ligands have low probability of blocking the Aβ_42_ interaction site in Na, K-ATPase in vitro, and therefore we eliminated NCI84171 and NCI298806 from the final ranking.

MD of the Na,K-ATPase:compound complexes provided three best potential inhibitors targeting its interaction with Aβ_42_. They were NCI617551 (represented in ZINC15 database by several entries: ZINC153412538, ZINC153412622, ZINC153412744, ZINC153412881, ZINC160342695, and ZINC160342850), NCI58783 (no ZINC15 entry), and NCI39921 (ZINC15 database entry ZINC150471868). NCI617551 was found to block the Aβ_42_ interaction interfaces in the three conformations of Na,K-ATPase after 50 ns MD simulations. The other two inhibitors blocked Aβ_42_ interaction interfaces in the two conformations of Na,K-ATPase following 50 ns MD simulations (Table 3).

The other two promising inhibitors that were been eliminated by MD were NCI39918 (ZINC15 database entry ZINC4430655) and NCI23128 (ZINC15 database entry ZINC150340408).

## 4. Discussion

Inhibition of the function of Na,K-ATPase by Aβ_42_ is one of the reasons for the impairment of the electrotonic properties of neurons in AD [19,40]. We previously established that the monomeric form of Aβ_42_ is able to bind to Na,K-ATPase, which contains the ubiquitous α1-subunit [18], and the inhibition of the enzyme occurs as a result of Aβ_42_ oligomerization on the Aβ_42_ molecule, which is primarily bound to Na,K-ATPase [10]. Thus, disruption of the binding of Aβ_42_ to Na,K-ATPase would prevent inhibition of the enzyme and the resulting impairment of neuronal properties at an early stage of AD. To search for the compounds disrupting binding, we had to define the interaction interface between Aβ_42_ and Na,K-ATPase.

According to our data, Aβ_42_ binds to the extracellular part of Na,K-ATPase [18]. Docking of Aβ_42_ to the extracellular region of the shark Na,K-ATPase crystal structure (PDB: 2ZXE [22]) showed that the beta-amyloid binding site is located at the junction of the α- and β-subunits [18]. However, the interaction interface between Aβ_42_ and human Na,K-ATPase has not yet been identified. In addition, during the catalytic cycle, Na,K-ATPase, according to the Albers–Post scheme, changes its conformation, passing from the state E1 to E2 through phosphorylated forms: E1-E1P-E2P-E2 [22]. Previously, data on the binding of Aβ_42_ to Na,K-ATPase [18] were obtained only in the buffer for measurements of activity in which both the E1 and E2 conformations of the enzyme were realized. The question of which conformation of the enzyme Aβ_42_ interacts with has long remained open.

Using molecular modeling, it was found that the interaction interfaces of Aβ_42_ with E1P and E2P conformations of human Na,K-ATPase, as well as with human Na,K-ATPase in complex with ouabain (OBN conformation), remained almost unchanged (Figure 2). These data were confirmed experimentally. The similarity between the values of the thermodynamic parameters of Aβ_42_ binding to the enzyme at the E1/E2, E1, E2, and E2P conformations (Table 2) unambiguously indicated that the interaction interface between Aβ_42_ and Na,K-ATPase did not undergo significant changes during conformational transitions of the enzyme.

These data represent a significant difference between the binding of Aβ_42_ and the specific ligand of Na,K-ATPase ouabain, which binds predominantly to the E2P conformation of the protein [39] and fixes the enzyme in the E2P-like conformation. The reason for this is the binding of ouabain in the gap between the transmembrane helices of the α-subunit of the enzyme [39,41], while Aβ_42_ binds mostly to the protein surface between the α- and β-subunits (Figure 2 and Figure S1). According to the molecular modeling data, the binding sites of ouabain and Aβ_42_ did not overlap, and remaining in complex with ouabain, Na,K-ATPase was also able to bind beta-amyloid (Figure 2). Experimental data confirmed this, demonstrating that the thermodynamic parameters of Aβ_42_ binding to Na,K-ATPase in complex with ouabain did not change (Table 2). Thus, ouabain binding did not affect the ability of Na,K-ATPase to bind Aβ_42_. Similarly, Aβ_42_ binding did not affect ouabain binding.

The obtained data are important for understanding the functioning of Na,K-ATPase in health and disease, indicating that even Na,K-ATPase fixed by ligands in one of its conformations will remain a target for Aβ_42_. Endogenous cardiotonic steroids can lead to the activation of signaling pathways through Na,K-ATPase [42], which acts as a receptor for these compounds. On the other hand, one can suggest that the binding of Aβ_42_ to Na,K-ATPase can also lead to the activation of the Src-dependent cascade [18]. Since ouabain and Aβ_42_ do not prevent each other from binding to Na,K-ATPase, it can be assumed that their joint binding to the enzyme is able to modulate the activation of signaling cascades. In a number of pathologies, an increase in the level of cardiotonic steroids is observed in the blood [43], and in the light of the obtained data, the effect of this phenomenon on the development of AD remains to be clarified.

When developing inhibitors of the interaction between Na,K-ATPase and Aβ_42_, it is also necessary to take into account that there are Aβ_42_ isoforms in the body system that differ in their pathogenic potential. Thus, the D7 isomerized isoform of Aβ_42_, which has a greater tendency to oligomerize in the presence of zinc, has greater cytotoxicity [8] and accelerates the development of amyloid plaques in a mouse model of AD [44]. At the same time, the phosphorylated form of pS8-Aβ_42_ does not have the ability to inhibit Na,K-ATPase and reduced the rate of plaque formation in a mouse model of AD [10]. As a result of comparison of the interaction interfaces of Aβ_42_, isoD7-Aβ_42_, and pS8-Aβ_42_ with human Na,K-ATPase, it became clear that the interfaces were almost identical. This is apparently due to the fact that in the peptide, these modifications are located at the N-terminus, which is less involved in the interaction with the enzyme. The obtained data are consistent with the fact that the thermodynamic parameters of Aβ_42_ and pS8-Aβ binding have similar values [10,18].

The similarity of the Aβ_42_ interaction interfaces with Na,K-ATPase in different conformations suggests that the performed screening of inhibitors for Na,K-ATPase in the E1P conformation have provided us with compounds that can prevent interaction with Aβ_42_ for other conformations of the enzyme. They could also be effective not only for unmodified Aβ_42_ but also for its phosphorylated and isomerized isoforms. Docking of the top 10 compounds obtained by screening to the E2P and OBN conformations of Na,K-ATPase followed by molecular dynamics confirmed that these compounds were potentially able to inhibit the interaction of Aβ_42_ with Na,K-ATPase in all three conformations as well. The most promising compound was NCI617551 (represented in ZINC15 database by several entries: ZINC153412538, ZINC153412622, ZINC153412744, ZINC153412881, ZINC160342695, and ZINC160342850). It overlapped the Aβ_42_ interaction interfaces in the three conformations of α1 Na,K-ATPase after 50 ns of MD. The other two inhibitors potentially blocking the Aβ_42_ interaction interfaces in two conformations of α1-Na,K-ATPase after 50 ns of MD were NCI58783 (no ZINC15 entry) and NCI39921 (ZINC15 database entry ZINC150471868).

## 5. Conclusions

According to our data, the interaction site with Aβ_42_ is located in a “gap” between the α- and β-subunits of human Na,K-ATPase. The structure of this site varies depending on the conformation of Na,K-ATPase; however, the exact residues participating in interaction remain the same. Computer modeling showed almost identical interaction interfaces for the three Na,K-ATPase conformations with Aβ_42_. This was confirmed by similar binding parameters of the Na,K-ATPase at different conformations with Aβ_42_. There was no pronounced difference between interaction interfaces for these conformations with the isomerized and phosphorylated isoforms of Aβ_42_ either. Consequently, beta-amyloid and its isoforms bind equally well to Na,K-ATPase in any of its states, while ouabain does not interfere with formation of Na,K-ATPase:Aβ complexes. This similarity in Na,K-ATPase interaction interfaces allowed us to cross-screen inhibitors for Na,K-ATPase at different states and find compounds that can potentially block interaction with beta-amyloid for all three analyzed Na,K-ATPase conformations. Obtaining and experimental testing of the identified inhibitors is scheduled for the subsequent phase of the project.

## Figures and Tables

**Table 1:** Interaction sites of E1P, E2P and OBN conformations of Na,K-ATPase with Aβ_42_ after docking and subsequent MD.

Conformation of the Na, K-ATPase	Interaction Sites with Aβ_42_ after Docking, a.a. Residues	Interaction Sites with Aβ_42_ after MD, a.a. Residues
E1P	α-subunit: 121–124 312–317 887–893 β-subunit: 84–87 216–222 270–273 287–290	α-subunit: 117–126 311–318 794 884–894 β-subunit: 82–87 91–111 196 217 266–273 287–298
E2P	α-subunit: 113–128 309–316 883–900 970–979 β-subunit: 82–107 197 216–226 267–273 285–290 294–303	α-subunit: 117–124 310–316 879 883 886–893 970–979 β-subunit: 83–85 91–107 217–218 268–273 287–290
OBN	α-subunit: 117–125 309–317 886–893 969–979 β-subunit: 82–87 96–107 216–221 271–273 287–290	α-subunit: 115–125 306–317 792–795 879 883 886–893 974 β-subunit: 2–87 91 100 103–107 216–218 273 287–290 294

**Table 2:** Thermodynamic parameters of Aβ_42_ binding to different conformations of Na,K-ATPase at 25 °C.

Na,K-ATPase State ^a^	K_a_,^b^ M^−1^	K_d_ ^c^ µM	∆H ^d^ kcal/mol	T∆S ^e^ kcal/mol	∆G ^f^ kcal/mol
E1/E2	7.7 × 10^5^	1.3	−2.54	5.48	−8.02
E1	3.7 × 10^5^	2.7	−1.54	6.02	−7.56
E2	4.9 × 10^5^	2.0	−2.21	5.55	−7.76
E2P	5.1 × 10^5^	2.0	−1.59	6.19	−7.78
OBN	8.3 × 10^5^	1.2	−1.21	6.87	−8.08

^a^ The measurements of Aβ_42_ binding to Na,K-ATPase in different states were performed in the four different solutions: E1/E2 state: 10 mM imidazole, 0.1 mM DTT, 130 mM NaCl, 20 mM KCl, 3 mM MgCl_2_, pH 7.5; E1 state: 10 mM imidazole, 1 mM EDTA, 3 mM NaCl, 0.1 mM DTT, pH 7.5; E2 state: 10 mM imidazole, 1 mM EDTA, 3 mM KCl, 0,1 mM DTT, pH 7,5; E2P state: 10 mM imidazole, 1 mM EDTA, 3 mM MgCl_2_, 3 mMTris/Pi, 0.1 mM DTT, pH 7.5; OBN state: 26 µM ouabain, 10 mM imidazole, 1 mM EDTA, 3 mM MgCl_2_, 3 mMTris/Pi, 0.1 mM DTT, pH 7.5. All measurements were performed three to four times at 25 °C. ^b^ K_a_—affinity constant; standard deviation did not exceed ±20%. ^c^ K_d_—dissociation constant; calculated as 1/K_a_. ^d^ ∆H—enthalpy variation; standard deviation did not exceed ±10%. ^e^ T∆S—entropy variation; calculated from the equation ∆G = ∆H − T∆S. ^f^ ∆G—Gibbs energy; calculated from the equation ∆G = −RTlnKa.

**Table 3:** List of the top-rated ligands obtained after virtual screening of the complete NCI Open Database using the Autodock Vina program for the Aβ_42_ interaction interface with the three Na,K-ATPase conformations. The complexes where the ligands (compounds) remained in the Na,K-ATPase sites forming the interaction interface after MD simulation are highlighted in green. The complexes where the ligands (compounds) entered the Na,K-ATPase cavity after MD simulation are highlighted in yellow. The complexes where the ligands (compounds) moved to the membrane after MD simulation are highlighted in red.

Compound Name in the NCI and ZINC15 Databases	Rating Number and Affinity to E1P, kcal/mol	Rating Number and Affinity to E2P, kcal/mol	Rating Number and Affinity to OBN, kcal/mol	Compound Chemical Formula
NCI39918 ZINC4430655	1 –9.4	4 –8.0	3 –8.6	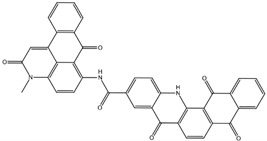
NCI610512 iodine (I) atom changed to hydrogen	2 –9.3	7 –7.4	5 –7.6	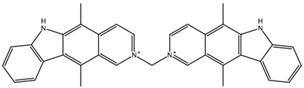
NCI686480 ZINC5542961	3 –9.1	3 –8.2	4 –8.1	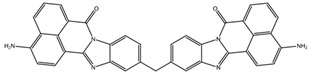
NCI39921 ZINC150471868	4 –8.9	1 –8.7	2 –8.7	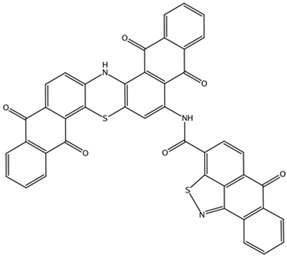
NCI617551 ZINC153412538 ZINC153412622 ZINC153412744 ZINC153412881 ZINC160342695 ZINC160342850	5 –8.9	2 –8.3	7 –7.4	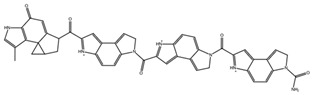
NCI84171 ZINC95857648	6 –8.7	5 –7.9	1 –8.9	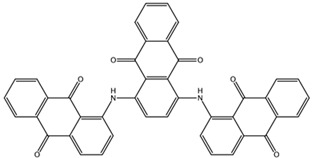
NCI298806 ZINC5390003	7 –8.7	10 –5.6	9 –6.7	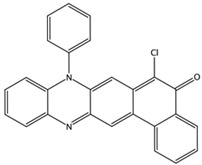
NCI23128 ZINC150340408	8 –8.7	8 –7.3	6 –7.6	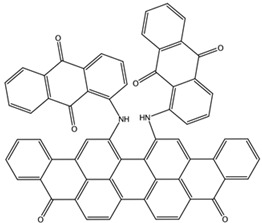
NCI688806 ZINC5543121 ZINC5543124 ZINC107044834 ZINC107044838 ZINC107044843 ZINC107044846	9 –8.7	6 –7.6	8 –7.4	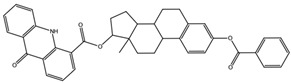
NCI58783 Na atom changed to hydrogen	10 –8.6	9 –6.5	10 –6.4	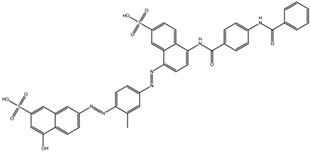

## Data Availability

Not applicable.

## Supplementary Materials

Supporting information can be downloaded at: https://www.mdpi.com/article/10.3390/biomedicines10071663/s1, Figure S1: Five complexes of Na,K-ATPase with Aβ_42_ obtained by fullblind docking as it described in methods section. Aβ_42_ peptides are colored green and the probable interaction sites are colored magenta; Figure S2: Interaction sites with Aβ_42_ on E1P Na,K-ATPase after 50 ns MD. Sites 119-AATEE-124, 310-SLILEY-315 and 887-RVDWDDR-893 on α-subunit and site 84-QKTEI-87 on β-subunit are concerned to present the main interaction interface and are colored with magenta. Other interaction sites 216-KRDEDKDKVG-226, 267-TMDTEIR-273 and 285-YSEKDR-290 on β-subunit are colored with cyan; Figure S3: The number of intermolecular contacts with three Aβ isoforms after targeted global docking of E1P (A), E2P (B) and OBN (C) conformations of α1 isoform of human Na, K-ATPase. The data for each conformation are summarized for 3 Aβ isoforms and about 30 complexes from docking.
