# Supplementary material for: Interaction Interface of Aβ42 with Human Na,K-ATPase Studied by MD and ITC and Inhibitor Screening by MD
Source: Biomedicines. 2022 Jul 11;10(7):1663. doi: 10.3390/biomedicines10071663 (PMC9313104; doi:10.3390/biomedicines10071663)
Supplement: Supplementary file 1 [file biomedicines-10-01663-s001.zip › biomedicines-1785576-supplementary (1)-1.pdf]

Supplementary

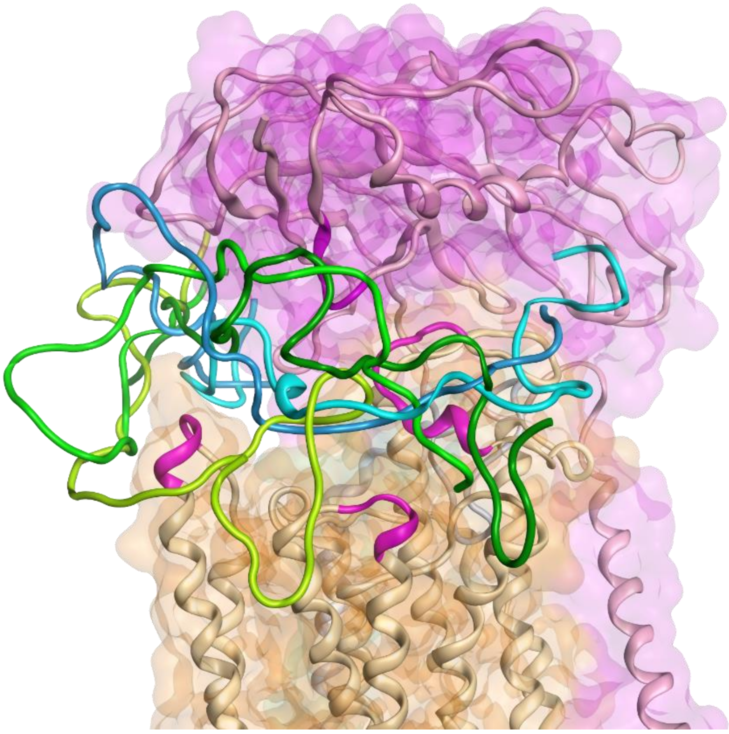

Figure S1. Five complexes of Na,K-ATPase with Aβ_{42} obtained by fullblind docking as it described in methods section. Aβ_{42} peptides are colored green and the probable interaction sites are colored magenta.

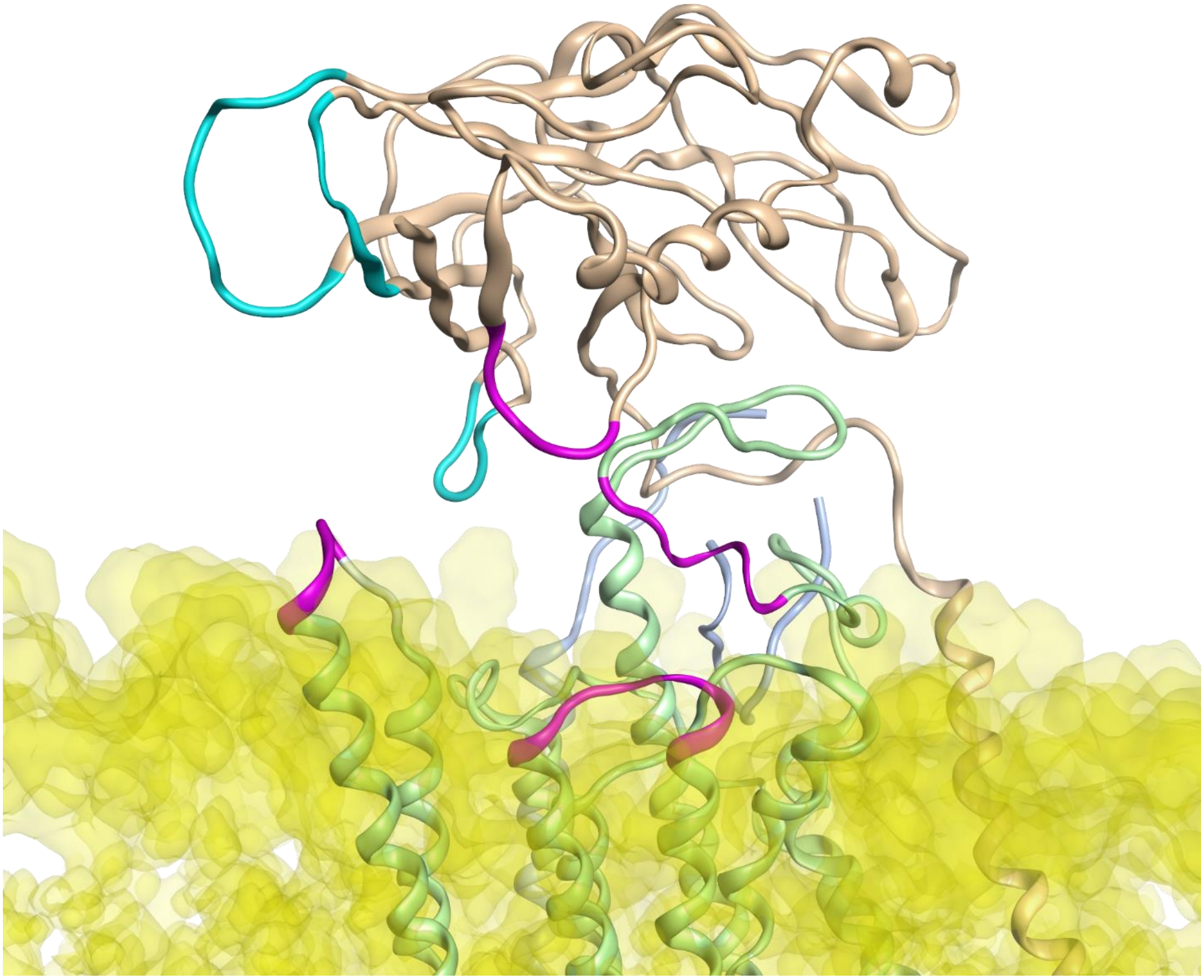

Figure S2. Interaction sites with Aβ_{42} on E1P Na,K-ATPase after 50 ns MD. Sites 119-AATEE-124, 310-SLILEY-315 and 887-RVDWDDR-893 on α-subunit and site 84-QKTEI-87 on β-subunit are concerned to present the main interaction interface and are colored with magenta. Other interaction sites 216-KRDEDKDKVG-226, 267-TMDTEIR-273 and 285-YSEKDR-290 on β-subunit are colored with cyan.

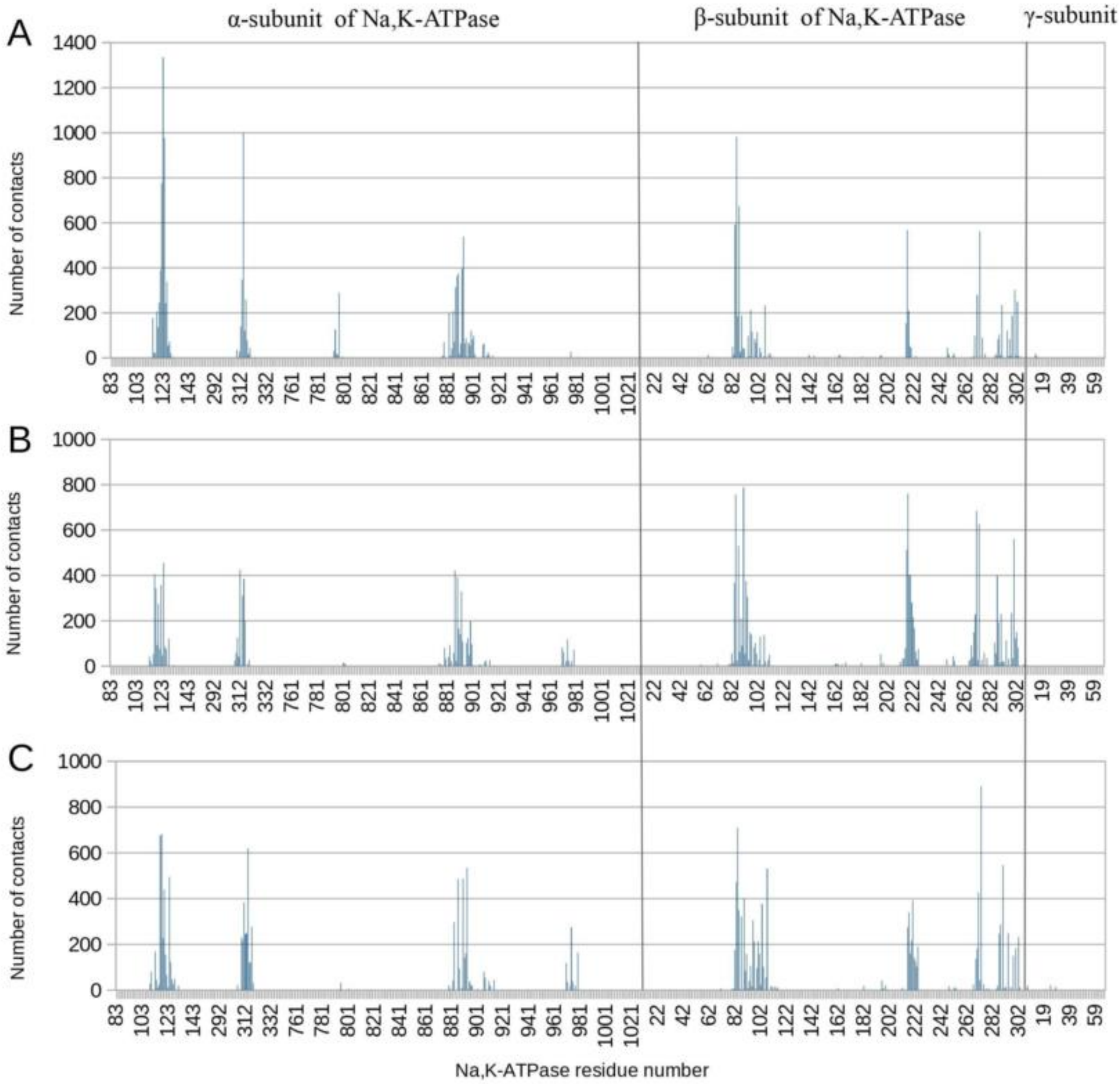

Figure S3. The number of intermolecular contacts with three Aβ isoforms after targeted global docking of E1P (A), E2P (B) and OBN (C) conformations of α1 isoform of human Na, K-ATPase. The data for each conformation are summarized for 3 Aβ isoforms and about 30 complexes from docking.
